# Post-Immune Antibodies in HIV-1 Infection in the Context of Vaccine Development: A Variety of Biological Functions and Catalytic Activities

**DOI:** 10.3390/vaccines10030384

**Published:** 2022-03-02

**Authors:** Anna Timofeeva, Sergey Sedykh, Georgy Nevinsky

**Affiliations:** 1 SB RAS Institute of Chemical Biology and Fundamental Medicine, 630090 Novosibirsk, Russia; sedyh@niboch.nsc.ru (S.S.); nevinsky@niboch.nsc.ru (G.N.); 2 Faculty of Natural Sciences, Novosibirsk State University, 630090 Novosibirsk, Russia

**Keywords:** HIV, antibodies, IgG, neutralizing antibodies, viral envelope, vaccine, HIV-1 vaccine, catalytic antibodies

## Abstract

Unlike many other viruses, HIV-1 is highly variable. The structure of the viral envelope changes as the infection progresses and is one of the biggest obstacles in developing an HIV-1 vaccine. HIV-1 infection can cause the production of various natural autoantibodies, including catalytic antibodies hydrolyzing DNA, myelin basic protein, histones, HIV-integrase, HIV-reverse transcriptase, β-casein, serum albumin, and some other natural substrates. Currently, there are various directions for the development of HIV-1 vaccines: stimulation of the immune response on the mucous membranes; induction of cytotoxic T cells, which lyse infected cells and hold back HIV-infection; immunization with recombinant Env proteins or vectors encoding Env; mRNA-based vaccines and some others. However, despite many attempts to develop an HIV-1 vaccine, none have been successful. Here we review the entire spectrum of antibodies found in HIV-infected patients, including neutralizing antibodies specific to various viral epitopes, as well as antibodies formed against various autoantigens, catalytic antibodies against autoantigens, and some viral proteins. We consider various promising targets for developing a vaccine that will not produce unwanted antibodies in vaccinated patients. In addition, we review common problems in the development of a vaccine against HIV-1.

## 1. Introduction

Since the first clinical detection of AIDS (acquired immunodeficiency syndrome) and the subsequent isolation of HIV (human immunodeficiency virus) in the early 1980s, the HIV epidemic continues to be one of the major health threats in the world, despite four decades of intensive research [1].

A high level of genetic variability in HIV-1 is one of the biggest obstacles in developing a safe and effective vaccine. Unlike many other viruses, HIV-1 is highly variable, with many subtypes and recombinant forms [2,3]. Low fidelity of HIV-1 reverse transcriptase leads to the rapid generation of mutants carrying base substitutions, insertions, and deletions. Combined with the addition and loss of glycosylation sites, this results in tremendous viral diversity [4,5,6]. A key source of genetic diversity is the viral gene env, which encodes envelope glycoprotein (Env), gp120, and gp41. As is known, gp120 and gp41 subunits on the surface of the virion are trimerized, form spikes, and together are involved in penetration into the target cell. Env has a complex conformation and undergoes significant rearrangements when binding to CD4 and coreceptors [7,8,9]. Glycans make up half the mass of the HIV-1 envelope glycoprotein, forming a topographic landscape that alters the availability of antibody (Ab) binding to the immunogen [10].

Env is also a major target for neutralizing antibodies (nAbs) [11,12]. The inability of the adaptive immune system to prevent and control infection is explained by the structural variability of the HIV-1 envelope. Protein gp120 consists of five relatively constant (C) regions and five highly variable (V) regions. Most adaptive responses target V-domain epitopes that mutate rapidly [13]. Cytotoxic T-lymphocytes and nAbs provide only temporary protection [14,15].

**Figure 1:**
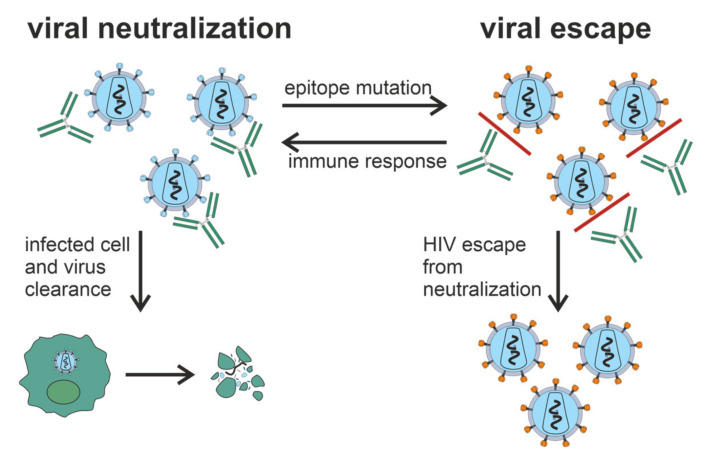
Generation of bNAbs, which neutralize the virus through binding to viral spikes and blocking the entry of the virus into sensitive cells such as CD4^+^ T cells, is related to the evasion of virus from the immune response due to the low precision of HIV-1 reverse transcriptase and rapid generation of viral mutants. Glycan shield, immunodominant variable loops, and conformational masking of key viral epitopes protect Env from Ab-response.

Progression of HIV-1 infection to AIDS occurs over 1 year to more than 2 decades [16]. Some HIV-infected patients develop Abs that can neutralize a wide range of HIV-1 strains [17,18,19]. HIV-1 has mechanisms preventing the production of broadly neutralizing antibodies (bNAbs) (Figure 1), including intense glycosylation of Env glycoproteins, instability of these glycoproteins, and conformational masking of receptor binding sites [20,21,22]. However, the mechanism behind the production of these bNAbs, and why it only occurs in some patients is not clear.

Despite the enormous diversity of HIV-1, there are described a few bNAbs such as b12 [23,24], 2G12 [24,25], 4E10 [26], 2F5 [24,27], VRC01 [28], PG9/PG16, 447-52D [29]. As shown in [30,31,32], b12 Ab isolated from the phage display library can neutralize about 40% of known HIV-1 strains. Another monoclonal antibody (mAb) HJ16, also neutralizes about 40% of viral isolates [33]. Most of the structures of such monoclonal bNAbs are resolved in complexes with Env [8,34]. These structures can be used to develop immunogens capable of eliciting bNAbs. Under the International AIDS Vaccine Initiative Neutralizing Antibody Consortium, a global program was established to identify HIV-infected individuals with broad and potent nAb activity as a potential source of new monoclonal bNAbs [20].

Although the detection of circulating autoantibodies (autoAbs) to self-antigens in patients infected by HIV-1 or other viruses does not necessarily reflect the presence of an autoimmune disease, such autoAbs can certainly complicate viral infections. These responses may vary due to the infection of autologous cells by the virus and their consequent targeting by pre-existing and/or induced autoAbs against the virus. Several natural autoAbs were described in HIV-infection [35]: Abs against small nuclear ribonucleoproteins (snRNP) [36], Abs to anticardiolipin (aCL) and antiglycoprotein 1 (aβ2GP1), anti-DNA, and antinuclear Abs [37,38]. Common to some viral diseases and autoimmune pathologies, catalytic Abs (Abzymes) hydrolyzing DNA [39] and a variety of proteins were described in HIV/AIDS patients: histones [40], myelin basic protein (MBP) [41], HIV integrase [42,43] and HIV reverse transcriptase [39], β-casein and serum albumin [39]. Such autoAbs may be beneficial for patients, as they expel autologous cells expressing viral antigens from the body. Moreover, one cannot exclude that HIV-infected patients produce natural Abs that recognize both microbial and autologous antigens.

Several vaccines in preclinical tests consisted of single coat proteins or in combination with other HIV-1 proteins and multiepitope synthetic peptides and polypeptides expressed by noninfectious vectors. Numerous HIV-1 vaccine candidates are directed to the induction of nAbs and/or cytotoxic T cells [44]. Despite the induction of robust immune responses, the recombinant glycoprotein gp120 VaxGen [45] and Merck gag/pol/nef (STEP) adenoviral vaccine [46] did not reduce the risk of infection. RV144 vaccine, consisting of the full-length gp120 protein and canarypox vector, reduced the risk of disease by 31% [47]. Similar to the case of the ineffective response caused by candidate vaccines, the pos-immune response, which follows HIV-1 infection, usually does not control the further spread of the virus [48]. According to the literature reviewed, some HIV-1-infected individuals develop hypermutated bNAbs through a postimmune Ab response. Therefore, advanced editing of Env-targeting B cell receptors (BCRs) may gradually lead to the generation of bNAbs. It remains unknown whether B cells naturally expressing Env-targeting BCRs can act as templates for the generation of bNAbs. Chronic HIV-1 infection may lead to the extensive BCR somatic hypermutation and the generation of Env-directed bNAbs [49]; knocked-in mice expressing unmutated common ancestors of BCR heavy and light chains show that HIV-1 Env immunization can boost the generation of bNAbs [50,51]. Recombinant Env immunogens may activate B cells that express the germline BCRs of bNAbs [52]. Vaccination of wild-type mice leads to affinity maturation of primary bNAb-engineered B cells and the generation of bNAb-memory and plasma cells [53].

The induction of a protective Ab response remains a top priority in HIV-1 vaccine development. Vaccines must be safe and not induce the development of Abs against autoantigens, which can exhibit autoreactive properties and trigger a cascade of various autoimmune reactions. It is known that immunization with one antigen can lead to polyreactive Abs, including those that bind to autoantigens.

In this review, we consider the whole spectrum of Abs described in HIV-infected patients, including nAbs (Section 2) specific to various viral epitopes, as well as Abs formed against various autoantigens (Section 3), including Abs with catalytic activity (Section 4) to these autoantigens and some viral proteins. In addition, we describe various approaches and difficulties on the way to the HIV-1 vaccine (Section 5) and the peculiarities of Abs production during COVID-19 in HIV-infected patients (Section 6).

## 2. Neutralizing Antibodies in HIV-1 Infection

Neutralizing Abs protect cells from a viral particle by binding it. These Abs surround the virus, after which the entire complex containing the virus is removed with the immune system. The induction of nAbs is a key goal of vaccination strategies [54,55].

During HIV-1 infection, almost all patients produce Abs to Env, but only a small fraction of these Abs can neutralize the virus [56,57]. Interestingly, nAbs against viral Env are produced within the first weeks of infection. Still, this early Ab response targets an autologous virus that circulates within each person and is ineffective against heterologous (unrelated) viruses [5].

Among the nAbs, a specific group of Abs neutralizing a wide spectrum of HIV-1 strains are distinguished. These broadly neutralizing antibodies (bNAbs) arise after several years of virus–antibody coevolution in infected patients [58,59,60]; bNAbs usually recognize conserved epitopes on the highly glycosylated envelope glycoprotein (Env) [61,62].

Several groups have shown that serum of 10 to 25% infected patients contains bNAbs [63,64,65]. Approximately 25% of HIV-infected subjects who have been infected for at least 1 year, without clinical symptoms of AIDS, and not taking antiretroviral drug therapy, showed moderate to extensive nAb responses [64,65,66].

Structurally conserved regions of the Env are functionally important for viral attachment and entry into target cells. These are the CD4 binding site and the coreceptor binding site located at gp120 [63]. It was assumed that viral epitopes, conserved among most viral strains, would generate cross-reactive Abs. However, for not fully understood reasons, these conserved viral epitopes were either weakly immunogenic or produced Abs with limited neutralizing reactivity [20,67].

Since the carbohydrates of Env glycoprotein may serve as a shield to evade the immune system, they can be used as targets for bNAb recognition [10]. Abs can bypass the glycan shield to access the surface of the viral protein. Rarely, bNAbs only recognize glycans; in contrast, glycan-dependent nAbs against HIV-1 targeting a combination of glycans and an underlying glycopeptide are not uncommon [68]. These glycopeptide-targeting Abs are often produced during natural infection [69].

Numerous works aim to study the specificity of Abs from HIV-infected patients and the relationship of these specificities with the breadth and efficiency of Ab-responses. At the same time, the study of epitope-specific nAbs is of particular interest since this data can be used in the development of vaccines [70]. The main challenge facing HIV-1 vaccine development is stimulating the bNAbs production; these issues are discussed in Section 5 of this review.

### 2.1. General Characteristics of nAbs

The first B cell response to HIV-1 infection appears within 8 days after detectable viremia [71]. After the next 5 days, circulating Abs against gp41 are detected, and after another 2 weeks, Abs against gp120 are detected, which primarily target the V3 loop. Autologous nAbs are developed over several months [70] and target variable HIV-1 regions by potent but particular molecules [5,72,73]. Over the years, Abs with neutralizing cross-specific potential developed in one third of chronically infected patients and targeted the more conserved regions of Env [63]. Heterologous nAbs appear in some patients 1 year after infection reach their peak after 4 years, with no increase after that [19]. High viral load is not a general predictor of Ab neutralization capacity since some patients with high viremia do not develop cross-neutralizing Abs [19,74].

The breadth of Ab neutralization is usually increased to the end of 2.5 years after infection [75]. In the long-term nonprogressor patients, all neutralizing Abs against gp120 were clonally associated with the antibody-diversifying process of somatic hypermutation [76]. B cells producing bNAbs have been shown to undergo several rounds of affinity maturation in germinal centers to achieve cross-neutralizing activity [77,78]. BNAbs develop over time and are maintained by chronic antigen exposure. Broadly cross-reactive nAb responses occur over several years, which means that Ab maturation response is required to target specific conserved viral epitopes effectively. Sustained viral replication under the conditions Ab-response leads to the continuous evolution of the viral Env to avoid nAb. This antigenic evolution can gradually focus the nAb response on less immunogenic but more conserved regions of Env [63].

Dysregulation of the immune system in the later stages of HIV-1 infection leads to a decrease of new Ab-responses. The ability to generate new autologous responses is declined after 2–3 years of infection, regardless of disease progression [79,80]. It is currently unknown whether nAbs play a role in suppressing viral replication during chronic HIV-1 infection. All we do know is that the virus successfully escapes nAbs responses [81,82,83]. Stimulating the bNAbs production after the vaccination is a promising and important task; however, we should not forget that producing polyreactive Abs that bind autoantigens might be an extremely undesirable side effect. For more details, see Section 3.

### 2.2. Antibodies against Env Epitopes

Several papers are devoted to mapping the Ab-specificity responsible for cross-neutralizing activity [84,85,86]. These nAbs recognize epitopes of monomeric gp120; in some cases, cross-neutralizing activity can be attributed to Abs recognizing linear epitopes in the membrane-proximal outer region (MPER) of gp41 [87,88]. It has been shown that the quaternary epitope at the end of the trimeric envelope structure, including loops V2 and V3, is often the target of cross-neutralizing Abs [17,32,89].

**Figure 2:**
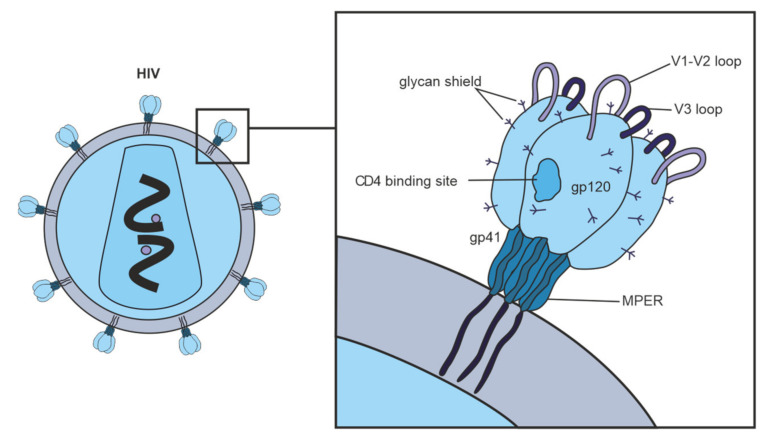
Vulnerable targets on trimeric glycoproteins of HIV-1 spike (gp120 and gp41). Widely neutralizing Abs target the CD4 binding site on gp120, the proximal outer membrane region of gp41, the glycan shield, and epitopes in variable loops 1, 2, and 3 on gp120.

Figure 2 shows vulnerable targets of HIV-1: nAbs react with the HIV-1 Env spike, which is composed of three highly glycosylated gp120 molecules, each noncovalently linked to a transmembrane gp41 molecule. To initiate entry of the virus into cells, gp120 binds to the CD4 receptor on the cell surface [90]. One of the structurally conserved regions of Env among the various isolates is the CD4 binding site on gp120, which is required for binding with the CD4 receptor on the surface of immune cells, such as T helper cells and monocytes. The second structurally conserved region of Env is the coreceptor binding site of gp120. Both the CD4 binding site and the coreceptor binding site are immunogenic, and numerous mAbs against these Env regions have been isolated from HIV-infected patients. However, most human mAbs against these regions cannot bind the native viral Env trimer and, therefore, cannot neutralize HIV-1 [63].

Abs against the CD4 binding site of gp120 can contribute to the overall cross-neutralizing potential of HIV-positive sera; in rare cases, the broad neutralizing activity of sera is almost exclusively due to such Abs [64,86,91]. Thus, the gp120 CD4 binding site is a promising target for vaccine development.

Abs to the gp120 coreceptor binding region also contributes to some sera’s cross-neutralizing activity [86]. It is assumed that the coreceptor binding site is only temporarily presented to the immune system during the fusion of the virus with the cell [70,92,93]. Known mAbs that bind to the gp120 coreceptor binding site do not neutralize HIV-1, presumably because they cannot efficiently access this site in the trimeric viral Env [93].

MPER of gp41 is highly conserved among various strains of HIV-1. MPER participates in virus fusion with the target cell and is the target of neutralizing human Abs 2F5, Z13, and 4E10 [26]. It was found that only a minority of the studied sera contained anti-MPER nAbs [63]. The fact that anti-MPER Abs can be involved in neutralizing HIV-1 is noteworthy since Abs to this hydrophobic region of gp41 can cross-react with lipid fragments on cell membranes of human cells. Therefore B cells producing such Abs can be autoreactive and likely are eliminated during B cell differentiation [94]. It is not surprising that nAbs directed against MPER rarely mediate broad and potent neutralizing activity [17,64,86].

We cannot exclude that a significant part of nAbs in sera are directed to unidentified regions of the viral Env. Possibly these are quaternary epitopes created by the association of three Env molecules that form a functional Env spike on the surface of infectious viral particles. It is also possible that carbohydrate molecules covering large surface areas of HIV-1 Env will form such unidentified epitopes [63].

Abs that bind to Env with high affinity would be ideal for neutralizing HIV-1. Still, the high variability of the Env gene and glycosylation patterns does not allow the development of a universal antigen that will solely stimulate the production of such Abs.

### 2.3. Hypervariable Domains gp120

HIV-1 gp120 contains five “hypervariable” domains. The first two domains (V1V2) include dramatic insertions and deletions and different glycosylation patterns [95]. The V1V2 region regulates the neutralization sensitivity of conserved epitopes such as the coreceptor binding site [8,96,97]. Among viruses of the B subtype, a high ratio of nonsynonymous and synonymous substitutions is characteristic of the V3 region, while in the C subtype, this region remains relatively conserved [98]. Abs against V3 play a minimal role in neutralizing primary viruses [31,99] because the V3 loop is clogged with trimeric Env [97,100,101]. In contrast to V1V2, the role of V4 and V5 in neutralization resistance is unclear, although these regions affect Env conformation and glycan packaging, thereby sterically limiting the availability of neutralization determinants [5,102,103].

Catalytically active Abs that hydrolyze gp120 are discussed in Section 4.1. To our knowledge, this protein is also unlikely to be a universal antigen for vaccine development.

## 3. Autoantibodies in HIV-1 Infection

Autoantibodies can interact with antigens specific to their organism. Pathogenesis of HIV/AIDS involves several factors, not a single virus-controlled destruction of CD4^+^ T cells [104]; among various pathogenic mechanisms there is autoimmunity [35] directed against lymphocytes [105,106,107], platelets [108,109] and peripheral nerves [110,111]. Up to 40% of HIV-1-infected patients are positive for autoAbs against red blood cells [112,113], which are involved in HIV-associated autoimmune hemolytic anemia [114,115].

Circulating Abs to small nuclear ribonucleoproteins are presented in the serum of HIV-infected patients [36]. Blood serum of HIV-1-infected patients contains Abs against anticardiolipin (aCL) and antiglycoprotein 1 (aβ2GP1), anti-DNA, and antinuclear Abs [37,38]. Exact frequency of autoimmune manifestations and their pathogenesis during HIV-1 infection remain unknown. Still, it was shown that anti-aCL Abs among HIV-1-infected patients is much higher than among patients with autoimmune diseases [116]. Content of these and similar Abs among HIV-1-infected patients before antiretroviral therapy ranges from 36% to 67% [37,117]. The presence of anti-aCL Abs in blood serum is not associated with any clinical manifestations of antiphospholipid syndrome, and it is not easy to explain why [118]. Moreover, spontaneous control of HIV-1 replication or subsequent after antiretroviral therapy is associated with lower production of anti-aCL Abs [119,120].

The physiological role of autoAbs generated during HIV-1 infection is not fully understood. Such autoAbs may be generated due to the “side function” of the immune system, directed to removal of autologous cells expressing foreign viral antigens (e.g., Env glycoproteins). Various HIV-1-related inflammations of the nervous system may lead to an increase of anti-MBP-Abs. General inflammation (especially in the case of chronic HIV-1-infection) may lead to the apoptosis-mediated or T cell cytotoxicity-mediated increase of extracellular DNA and its complexes with histones, as well as consequent production of anti-DNA and antihistone Abs. Due to the variability of HIV-1 surface antigens and large viral load in some patients, such virus-specific Abs may be polyreactive [121,122] and recognize both viral and autologous antigens. Autoreactive Abs are often encoded by intrinsically autoreactive VH family members such as VH4-34 [123], VH1-69 [124], and others and also may provide broad neutralizing activity [125].

Among autoAbs in HIV/AIDS, Abs against DNA, histones, and MBP should be discussed separately, especially those that bind these substrates and catalyze their specific hydrolysis.

## 4. Catalytic Antibodies in HIV-1

Autoimmune reactions and the appearance of autoAbs in HIV/AIDS and some other viral infections may be associated with the activation of polyclonal B cells, molecular mimicry between viral and/or microbial antigens and host antigens [126,127], abnormal expression of immunoregulatory molecules, and anti-idiotypic network [38,128]. One can propose several physiological functions of the hydrolysis of DNA, histones, and MBP by Abs. We suggest that the most relevant function of such catalytic Abs is the elimination of autoantigens formed due to the destruction of infected cells from the blood. However, we cannot exclude the possibility that such autoAbs may also explain some of the observed autoimmune pathologies found in patients with HIV/AIDS.

Activation of B-lymphocytes in HIV-1-infected patients leads to the production of Abs to viral components and autoAbs to various components of human cells [38]. It has been shown that IgG and/or IgM of patients with AIDS hydrolyze not only autoantigens as DNA [129], MBP [41], histones [40,130,131,132], but also viral enzymes HIV integrase [42,43,133,134] and HIV reverse transcriptase [39] and corresponding peptides. Interestingly, IgG of HIV-1-infected patients efficiently cleaves five human histones (H1 [41,130], H2a, H2b [131], H3, and H4 [132]).

Some IgG preparations from patients with lymphadenopathy exhibit exceptionally high proteolytic activity, and as the disease progresses to AIDS, the percentage of patients with detectable IgG activity increases and reaches 100%. Casein-hydrolyzing Abs have been found in the serum of 95% of AIDS patients and have been shown to have catalytic activity similar to serine protease [39].

It is unlikely that the HIV-1 vaccine might stimulate the generation of such autoAbs in the body of healthy patients after immunization or that vaccination sometime later will promote the generation of these Abs after HIV-1 infection. However, as shown in the following subsections, the production of some catalytic Abs is highly undesirable.

### 4.1. Catalytic Antibodies Cleave gp120

Hydrolysis of gp120 is possessed by V domains of the heavy (VH) and light (VL) chains of natural IgG, IgA, and IgM [135,136]. A rare light chain of Abs, isolated by phage display, can bind and hydrolyze gp120 independently of the heavy chain [137]. The most described Abs catalytic sites are located in the VL domains [138].

Long-term HIV-1 infection for 5 years is associated with a modest increase in catalytic IgA against gp120 [136]. No accumulation of such Abs was observed at the earlier stages of disease (6 months) [139].

Using synthetic peptides of gp120, it was shown that amino acid residues 421–433 are required for binding to the host CD4 receptors [140]. Epitope 421–-433 is relatively conserved across different strains of HIV-1 since IgM [137], IgA [136], and free light chains [141] of HIV-1 infected patients hydrolyze peptide bond between amino acids 432–433 located within this epitope.

According to the fundamental principles of biocatalysis and the data found in the available literature, Abs that hydrolyze gp120 are unlikely to possess broad neutralizing activity. As is well known [142], catalysis and binding occur on opposite ends of a “seesaw” (in order for bNAb to exhibit neutralizing activity, effective binding is required, making it incompatible with efficient catalysis, for which highly specific binding is highly undesired). Moreover, a level of catalysis detectable in vitro or in vivo is difficult to imagine for the tightly binding Abs. We suppose that the production of Abs that hydrolyze gp120 is unlikely to be beneficial for patients.

### 4.2. Antibodies Hydrolyze HIV-1 Integrase, Reverse Transcriptase, and Corresponding Oligopeptides

IgG and IgM were isolated from the blood serum of HIV-1-infected patients and subsequently separated on columns with immobilized reverse transcriptase or integrase, respectively, specifically hydrolyzed only recombinant viral reverse transcriptase [39] or integrase [42,43]. Abs that specifically hydrolyze these HIV enzymes were of potential interest in developing new anti-HIV-1 drugs for many years.

Nearly 40 sites of HIV integrase cleavage by Abs isolated from HIV-1-infected patients were found. Most sites are located in seven known immunodominant integrase sequences [133]. Two 20 mer oligopeptides corresponding to immunogenic integrase sequences contained 9 to 10 clusters of major, moderate, and minor cleavage sites [133,143]. Each individual Ab preparation from HIV-1-infected patients had a distinct ratio of hydrolysis sites [133].

Anti-integrase Abs first cleave intergrase accumulating long fragments, further degrading these long intermediates and the formation of very short products [133]. It has been shown that anti-integrase Abs of HIV-1-infected patients effectively hydrolyze specific oligopeptides and nonspecifically tri- and tetrapeptides [133,143]. Moreover, catalytically active anti-integrase Abs efficiently hydrolyzed oligopeptides corresponding to the immunogenic sequences of reverse transcriptase [134] and one oligopeptide corresponding to the immunodominant region of MBP [144].

Catalytic Abs are highly specific for globular proteins, but some of them can effectively cleave nonspecific tri- and tetrapeptides [134,145,146]. Catalytic sites of proteolytic Abs are usually located in the VL, while the heavy chain is responsible for specific antigen recognition and increasing antigen-Ab affinity. It is not surprising that the active center of anti-integrase Abs is located on the light chains [42,43].

The affinity of Abs to short oligopeptides is 100–1000 times lower than for the corresponding globular proteins [134,145,147]. Therefore, depending on the amino acid sequence, hydrolysis of oligopeptides with Abs is less specific or completely nonspecific [133,143].

The physiological function of the hydrolysis of enzymes involved in HIV nucleic acid metabolism (i.e., reverse transcriptase and integrase) by catalytic Abs is unknown. It is postulated that Abs can penetrate the cell nucleus and function as proteases therein [148,149], but these processes have never been shown in vivo in the case of HIV-1 infection. According to some reports, the generation of such catalytic Abs may positively affect HIV patients [150]. Since the production of catalytic Abs can be stimulated by immunization with transition states of a chemical reaction, the use of substances such as immunogens may be of particular interest in designing HIV-1 vaccines. Unfortunately, no single case was found where/wherein such catalytic activity was shown for the bNAbs.

### 4.3. Antibodies Hydrolyze DNA, Histones, and Myelin Basic Protein

Relative DNase activity of Abs isolated from the blood serum of HIV-1-infected donors varies significantly from patient to patient, but 96% of the preparations show a detectable level of DNase activity [129], which is an intrinsic feature of Abs [39]. Abs against H1, H3, H4, and MBP possess enzymatic cross-reactivity [151]: anti-histone H1 IgG hydrolyze H1 as well as MBP, and vice versa, anti-MBP Abs hydrolyze this H1 histone [41]. Similarly, IgG against H2a and H2b histones efficiently hydrolyze these histones and MBP, and anti-MBP Abs cleave MBP, H2a, and H2b histones, but not other control proteins [151]. Near 100% of IgG of HIV-infected patients effectively hydrolyze between one and five human histones [40].

Table 1 presents data on all currently known to authors on catalytic Abs in HIV/AIDS.

It is challenging to explain binding polyspecificity and catalytic polyreactivity of natural anti-MBP and antihistone Abs in HIV/AIDS only by the similarity of these antigens. It is known that anti-MBP and antihistone Abs play a negative role in the pathogenesis of systemic lupus erythematosus and multiple sclerosis [153,154,155]. These catalytic Abs may possess a wide range of physiological functions, such as clearing the blood of HIV-infected patients from excess autoantigens formed due to cell destruction. We assume that these catalytic autoAbs are highly undesirable, and their possible generation should be strictly avoided when designing prospective vaccines.

## 5. HIV-1 Vaccines: Production of Neutralizing Antibodies

Current HIV-1 prevention and treatment strategies include using antiretroviral drugs for pre-exposure prophylaxis and antiretroviral therapy. This made it possible to transform HIV-1 from a life-threatening disease to a manageable chronic disease [156]. However, drugs are expensive, require strict dosing to be effective, and cause side effects. Some HIV-infected patients develop drug resistance; furthermore, drug access remains a significant barrier, especially in low- and middle-income countries. Thus, a preventive vaccine remains a central component of a multidimensional strategy to end the HIV-1 epidemic. However, developing an effective vaccine against HIV-1 has proven to be a daunting task. To date, there is still no single approved HIV-1 vaccine, and only one promising clinical trial (the “Thai” trial of RV144) has shown a modest 31% efficacy [157].

HIV-1 replicates chronically in the host and evades an Ab response, unlike other viral pathogens. Immunity evasion and large genetic variation among HIV-1 strains are significant obstacles to vaccine development. The use of vaccines should provide generation of Abs that can adapt to the Env glycan screen and bind to various viral strains to offer complete protection [158,159]. An ideal HIV-1 vaccine would elicit serum Abs that are highly effective and can prevent viral infection at low concentrations and have broad action—making a high percentage of hard-to-neutralize viruses inactive [160].

HIV-1 vaccine should induce both humoral and cellular immunity. Abs neutralizing the virus will provide the first layer of defense by preventing infection of host cells as the virus enters the body. When some virions start to escape nAbs, cytotoxic CD8^+^ T cells will provide a secondary level of protection, eliminating the earliest infected cells preventing the generation of the latent reservoir of HIV-1-infected cells [161]. Table 2 shows all current completed HIV-1 vaccine studies.

### 5.1. HIV-1 Vaccine Development Strategies

**Figure 3:**
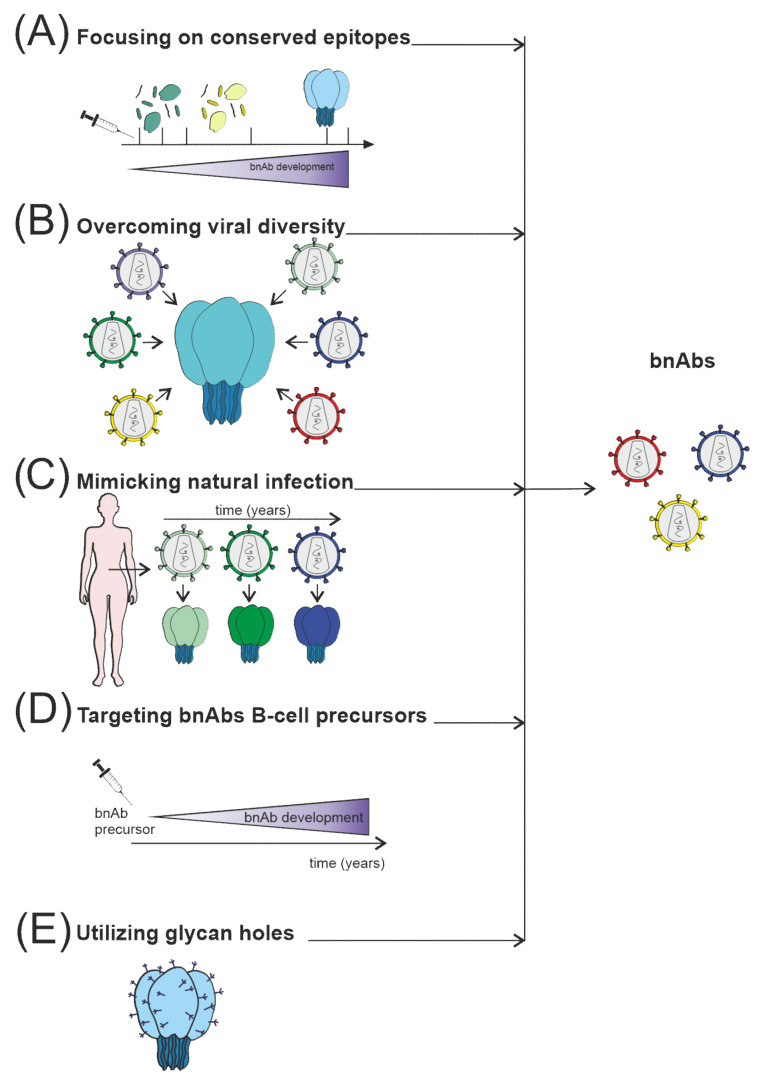
Several strategies are currently used to stimulate nAb responses towards bnAb generation, which consist of (**A**) directing of Ab response to conserved sites, for example, using fusion peptides; (**B**) use of mosaic or consensus antigens to overcome the viral diversity of circulating HIV-1 worldwide; (**C**) clonal immunogens resembling the evolution of the virus in HIV-1 infected individuals modeling natural infection; (**D**) targeting immunogens to putative bnAb germline progenitors, followed by immunization leading to the affinity maturation pathway to bnAb developm€; (**E**) selective removing of glycans to focus Ab responses to a specific site of interest, such as CD4 binding site immunogens.

The induction of a protective Ab response remains the main priority in developing HIV-1 vaccines [162]. Unfortunately, most current HIV-1 vaccine candidates do not elicit nAbs against most circulating viral strains. To replicate chronically in the host, the HIV-1 uses several mechanisms to defend itself against Ab recognition: Env glycoprotein is protected by the shield of glycans. Different variations occur in immunodominant loops, and key viral epitopes are due to the conformational lability [5,21,56] (Figure 3). These defense mechanisms, although very effective, have vulnerabilities; information on the precise location and molecular structure of these vulnerable regions can be useful for the rational design of improved vaccine immunogens.

Another line of research is related to the stimulation of the immune response on the mucous membranes. It has been shown that bNAbs can neutralize most virus strains in a cross-over manner and provide reliable mucosal protection in a monkey model [163,164,165].

In addition, induction of cytotoxic T cells has become a favored strategy for HIV-1 vaccination. Cytotoxic T cells can lyse infected cells and hold HIV-1 after infection. However, T cells do not inactivate free viral particles. Moreover, cytotoxic T cell response cannot adapt to the escape mutants resistant to vaccine-induced immunity [166].

Strategies for developing vaccines based on Abs to the variable domains of gp120 were abandoned, since such Abs only provide strain-specific neutralizing activity [167].

Recent discoveries have generated interest in “non-neutralizing” Abs that cannot directly inhibit the entry of the free virus into target cells but exhibit antiviral activity mediated by the Fc region of the Ab molecule. These Ab effector mechanisms include complement binding and viral lysis, phagocytosis of Ab-coated virions, and Ab-dependent cellular cytotoxicity [168,169,170].

Today, we do not understand the regulatory pathways of B cells completely. Additional information on the mechanisms responsible for migration, selection, and differentiation of B cells can be valuable to target appropriate Env epitopes to the specific pathways of B cell induction [67]. Most studies of humoral responses to HIV-1 infection have examined Abs, the final product of B cell response. Relatively few studies have examined the immunopathogenesis of B cells. However, several fundamental questions remain unanswered.

### 5.2. Immunogen Structure Design

HIV-1 predominantly induces non-neutralizing or strain-specific Abs during the first months after infection [5,70]. It was found that approximately 10–20% of HIV-1-infected patients had bNAbs after a few years of disease [17,63,170], which is the humoral immune response that a vaccine should elicit.

Viral epitopes conserved among most viral strains are more likely to generate cross-reactive Abs. In this regard, research is focused on small numbers of human mAbs isolated from HIV-1-infected individuals that possess cross-reactive neutralizing activity [56,81]. However, these conserved viral epitopes are weakly immunogenic or produce Abs with limited reactivity. Minor structural changes may improve specific binding [20,67]. The crystal structure of b12 mAb bound to the CD4 receptor binding site of the gp120 molecule provides insight into how nAbs gain access to the functionally conserved regions of the Env glycoprotein [34]. A better understanding of the gp120 and gp120-gp41 complexes structure and binding of Abs to the Env glycoprotein may provide new insights into the development of vaccines. Efforts to stabilize gp120 in a more immunogenic form and create a scaffold of conservative neutralizing epitopes may lead to more effective Ab responses [67].

One of the promising strategies for generating bNAbs after vaccination is the use of naïve B cells with long heavy chain complementarity-determining regions (HCDR3). These long HCDR3s are generated at the stage of V(D)J recombination [171] and are selected by Env, which results in the production of bNAbs targeting conserved epitopes [172]. BG18 is one such example [173]: it contains HCDR3 that does not have insertions or deletions since it can be easily induced in vivo [174]. Other long HCDR3s are described in rare B cells of HIV-1-naïve individuals, which binds to gp120 and neutralize the virus [175]. Interestingly, polyreactive BCRs may also contain long HCDR3; some other data suggests that bNAbs are intrinsically poly- or autoreactive and might be products of aberrations of immune tolerance controls [176].

Detailed molecular and immunological studies of the autologous neutralization response would improve the understanding of viral determinants vulnerable to Ab attack. Of particular interest are epitopes that direct autologous nAbs in HIV-1-infected patients. These epitopes can be highly variable, but apparently, there is no limit to the degree of variability that HIV-1 can exhibit in these regions [177,178].

One of the problems of induction of bNAbs is the limitation of suitable immature B cells, which may express BCR encoded by intrinsically autoreactive VH genes or contain long HCDR3 [179]. Moreover, these cells may not be ready for activation even by optimally engineered HIV-1 immunogen due to the B cell tolerance [180]. As a result of their intrinsic autoreactivity, these B cells may undergo negative selection in germinal centers [181]. Finally, unrestricted accessibility of an optimally engineered HIV-1 immunogen to these B cells may trigger severe autoimmunity, which is highly undesirable.

### 5.3. Vaccination Inducing Immune Response at Mucous Membranes

Mucosal tissues of the gastrointestinal tract and vagina are the main reservoirs for the initial replication and reproduction of HIV-1, as well as sites of rapid depletion of CD4^+^ T cells [182]. Such viral reservoirs are considered significant difficulties in eradicating HIV-1 in infected hosts. A successful vaccine should prevent the creation of these reservoirs at a very early stage of HIV-1 infection [183]. Protection of mucous membranes from HIV-1 is mainly provided due to the Abs. Vaccination is not necessarily resulting in extremely high levels of nAb to protect the mucosal surface. Still, the Ab response must be robust, and nAb must cross-react with the genetically diverse spectra of HIV-1 [184].

IgA is the predominant Ab isotype in most human mucosal secretions [185]. It has been reported that dimeric IgA applied rectally can not only protect against the rectal route of HIV-1 infection but be more effective than the corresponding IgG [186]. Several studies on humans have also found a correlation between high levels of secretory IgA (SIgA) and protection in high-risk individuals who remain seronegative [187,188].

Systemically delivered viral vectors can induce a mucosal immune response against HIV, especially in the intestinal, rectal, and genital mucosa [189,190], but the strength of these responses is usually low. After systemic immunization, adenoviral vector vaccines induce low mucosal immune responses [184]. However, very few vaccines provide mucosal immunity against any infectious disease [191].

It is known that nasal introduction of vaccine results in the generation of Abs in the respiratory system mucosa and saliva and nasal secretions, but not in the gut or vagina [192]. Vaginal vaccination should be administered on specific days of the cycle and therefore might not be optimal for general use [193]. Protective immunity against HIV-1 was demonstrated after oral administration of the gp120-based vaccine [194]. Since most homosexual and vertical transmission of HIV-1 occurs via the gastrointestinal tract [193], HIV-1 vaccines, delivered to the mucosal tissues, are very effective in generating gut immunity, making the oral way of HIV-1 mucosal vaccine delivery highly perspective.

One promising approach to stimulate mucosal immunity after vaccination is virus-like particle (VLP) vaccines. VLP are genomic-free viral particles (pseudovirions) produced by the spontaneous assembly of viral capsid proteins. They are similar in size and structure to intact virions, but they do not replicate and are pathogenic. These immunogens can be administered as purified DNA particles or plasmids expressing viral proteins required for in vivo VLP formation [195,196]. Several successful VLP vaccines have been developed against sexually transmitted HPV (human papillomavirus) and tested in human trials (flu), demonstrating the potential efficacy of VLP as candidates for an HIV-1 vaccine [197,198].

Since the digestive tract is permanently faced with a substantial antigenic load, the problem of efficient induction of mucosal Ab response and avoidance of mucosal tolerance after antigen introduction is crucial. Anti-HIV-1 vaccination of immunologically naïve patients may have an undesirable effect in decreasing cytotoxic T lymphocyte immunity. Since the other mucosal vaccines (against the poliovirus, influenza, and other viruses) possess protective effects as the result of Abs generation, but not via the cytotoxic T lymphocytes, this problem is especially relevant in the case of HIV-1 vaccines [199].

The human gut contains various commensal microbes, its disruption after oral vaccination could, in theory, lead to severe diseases, such as inflammatory bowel disease. Use of adjuvants or other vaccine components [200] may attenuate intestinal tolerance, increase intestinal immunity [201], and lead to the inflammatory response or dysbiosis. These problems should be considered during the development of prospective oral HIV-1 vaccines. Since eight oral vaccines against cholera, salmonella, influenza virus, poliovirus, and rotavirus are currently licensed for use in humans [202], this brings closer the perspective of the development of the HIV-1 oral vaccine.

### 5.4. Covalent Epitope-Based Vaccines

Although the virus mutates rapidly, it must retain specific surface protein epitopes to maintain its infectivity. Inducing a robust immune response against structurally conserved epitopes essential to the viral life cycle is a logical route to a vaccine development that will be effective worldwide and minimize the possibility of viral mutants’ escape. The target site should be expressed on the surface of free virions in a form that is sterically accessible to Abs. The main problem is that the vulnerable epitopes of HIV-1 are weakly immunogenic. Peptide immunogens with sequences identical to the linear conserved epitopes of gp120 can be easily synthesized, but the conformation of such peptides may also differ from that of the native epitope. Conformational states of the non-native peptide induce Abs with useless, non-neutralizing specificity. Conformational mimicry of discontinuous epitopes taken from conserved regions of gp120 has been hampered by the limitations of modern physicochemical methods for accurately assessing the structure and dynamics of a protein binding to Abs. Therefore, until recently, no promising epitope-based vaccine candidate has emerged [48].

Rare mAbs against gp120 and gp41 have a relatively broad neutralizing activity, for example: b12 [34], 2G12 [203] and 2F5 [27]. Abs common to these mAbs are not detectable in the polyclonal Ab mixture present in the blood of HIV-infected humans or animals immunized with experimental immunogens. Certain coreceptor binding site epitopes that are sterically inaccessible to Abs become exposed after gp120 binds to CD4. The temporary presentation of such epitopes on the surface of the gp120 protein limits their effect on Abs generation. It is encouraging that the exposed conserved epitope at the coreceptor binding site of the CD4-independent form of gp120 is immunogenic enough to induce extensive neutralization of Abs [48]. CD4 binding site 421–433 region is the notorious Achilles heel of the virus. Production of Abs to this area by traditional B cell differentiation pathways does not occur, but when anti-421–433 Abs appear, they neutralize various viral strains with exceptional efficiency [204,205]. Despite the difficulties described above, we suggest that the covalent epitope-based vaccines are promising direction of HIV-1 vaccine development.

### 5.5. HIV-1 mRNA Vaccines

The SARS-CoV-2 pandemic introduced the world to a new type of vaccine—mRNA encapsulated in lipid nanoparticles. mRNA-based vaccines rely on the production of individual immunogens by the host cells, which in turn are targets for Ab responses and cytotoxic T cells [206]. Traditional subunit vaccines deliver immunogens in a protein that degrades immediately after injection. In contrast, mRNA-based vaccines induce weekly production of immunogens in situ [207], increasing the possibility that rare bnAb progenitor B cells will become activated and proliferate after immunization. Prolonged production of mRNA-encoded immunogens in vivo works much like antigen release via an osmotic pump. Slow antigen delivery increases the activity of the germinal center and the induction of HIV-1 nAbs [208].

Strategies that increase the immunogenicity of Env antigens increase the chance of activation of rare B cells carrying germline progenitors with bnAb potential. One approach to improve the immunogenicity of Env is to map the Env immunogens onto arrays of particles such as nanoparticles or virus-like particles [209,210]. An antigen multimerization technique that lends itself to the mRNA platform of the vaccine is to encode a self-assembling scaffold protein, such as a ferritin nanoparticle, in addition to the Env immunogen [211]. Multimerization of Env on ferritin nanoparticles improves the immunogenicity of Env in animal models [212,213], thereby enhancing the activation of rare and/or anergic germline precursors of bnAb.

Although research and development innovations have led to rapid advances in mRNA vaccines over the past few years, many unresolved questions and challenges remain in developing an mRNA-based HIV-1 vaccine. There is no comprehension of the mechanisms of mRNA immunogenicity. Mainly, which cells express mRNA and produce the encoded immunogen? How do the immunogens encoded by mRNA interact with various immune system cells? What are the kinetics and magnitude of expression of the immunogen encoded by mRNA in vivo, especially with complex HIV-1 antigens? The answers to these questions will provide significant guidance for developing and evaluating mRNA-based HIV-1 vaccines [214].

Another problem is that correctly folded Env trimers can be purified before immunization in vaccines containing protein subunits. However, when Env immunogen is delivered as mRNA, it is impossible to purify protein trimers. To overcome the problem, the strategy of Env stabilization in cells needs to be developed [215,216,217].

Another critical issue in developing an HIV-1 immunogen for mRNA vaccination is associated with the inevitable post-translational modification in the host cell [218]. At an early stage of immunogen design, it is essential to consider the shape of the protein product encoded by the mRNA produced by the host cells. The global success of the SARS-CoV-2 mRNA vaccines allows us to dream that this perspective approach can design an HIV-1 vaccine, stimulating the generation of bNAbs, considering the limitations described above.

## 6. Immune Exhaustion and Antibody Response

Immune exhaustion is the loss of effector functions and proliferative capacity of memory T cells [219]. The process of immune exhaustion in the context of persistent viral infections was first described in a mouse model of lymphocytic choriomeningitis (LCMV). It has been shown that LCMV-specific CD8^+^ T cells are preserved during chronic infection but are not cytotoxic [220]. Subsequently, T cell exhaustion has been described in patients with chronic viral infections such as HIV-1, hepatitis B, and hepatitis C [221,222,223].

Immune exhaustion was described for both CD8^+^ and CD4^+^ T cells [224,225]. Antigen-specific exhaustion of effector and memory cells includes a gradual loss of effector function and proliferative capacity, which progress until such cells are eliminated [226]. Exhausted CD8^+^ T cells significantly differ from terminally differentiated or memory CD8^+^ T cells: they secrete fewer cytokines and are characterized by cell surface expression of inhibitory receptors such as PD-1, CTLA4, Tim3, TIGIT, CD160, and LAG-3 [227,228,229]. Such surface expression of inhibitory receptors increases upon activation and limits the overactivation of T cells, providing the so-called immune checkpoints (IC) [224].

HIV-specific CD8^+^ T cells upregulate PD-1 expression in untreated infection as their effector function declines. PD-1 is also activated on HIV-specific CD4^+^ T cells, while in vitro blockade of PD-1 restores the proliferative nature of these cells lost after exhaustion [230]. Expression of CTLA-4 by CD4^+^ T-cells was shown for HIV-1 infected patients, while in vitro blockade of CTLA-4 leads to a significant increase in proliferation [231]. Interestingly, specific IC markers on CD4^+^ T cells are associated with higher levels of HIV-1 DNA, suggesting that these ICs somehow contribute to latency in these subsets [232,233].

The “shock and kill” strategy for HIV-1 consists of clearing the HIV-1 reservoir in resting CD4^+^ memory T cells [234]. During the “shock” phase, drugs called latency-reversing agents are used to reactivate viruses that persist latently in the cell due to the increase in the expression of viral gene products. In the “kill” phase the infected cells are destroyed [235]. Hypothetical treatment of HIV-1 would work in two potential ways: by enhancing the effector function of HIV-specific CD8^+^ T cells and reversing the HIV-1 latency period. In animal models, the introduction of IC blockade has a significant effect: for example, blockade of PD-1 leads to a rapid expansion of SIV-specific CD8^+^ T cells with increased functionality [236], and blockade of CTLA-4 leads to an increase in plasma viremia and activation of T cells [237]. Prevention of the inhibitory signals will lead to increased gene expression and subsequent production of viral proteins, making the cells “visible” to both the immune system and antiretroviral treatment, as well as becoming susceptible to virus-mediated cytotoxicity [238].

Anti-IC Abs expressed on infected cells can be used for blocking IC [239]. Abs against the IC molecules block their inhibitory action, preventing exhaustion of CD8^+^ T cells [240]. In untreated monkeys infected with SIV [236,241], administration of anti-PD-1 Abs expanded and increased the functionality of virus-specific CD8^+^ T cells, significantly reduced RNA survival in the blood plasma, prolonged survival [236], and resulted in a decrease in immune activation markers [241]. Introduction of Abs against PD-L1 reduced HIV-1 replication and increased the number of CD4^+^ T cells in untreated HIV-infected humanized mice [242].

Anti-PD-1 and anti-PD-L1 mAbs improve specific CD8^+^ responses for HIV-1 and show immune-mediated toxicity. Thus, IC blockade is a potential tool for reversing immune exhaustion and could be a component of an HIV-1 treatment strategy [239].

## 7. COVID-19 and HIV-1

There is little evidence on the contribution of HIV-1 to previous epidemics of respiratory viruses. HIV-1 is associated with a higher risk of severe respiratory infections, including seasonal influenza [243,244]. However, the contribution of HIV-1 infection to outcomes during the 2009 H1N1 influenza pandemic was unclear. There would be no substantial evidence that HIV-infected individuals were at increased risk of infection or had worse outcomes unless they were in advanced immunosuppression [245]. HIV-1 infection was not associated with increased disease severity during previous SARS and MERS outbreaks; there were only a few reports of mild illness among people living with HIV-1 [246,247].

The SARS-CoV-2 pandemic has become the greatest threat to global health in the modern era. It should be recognized that HIV-1/AIDS and COVID-19 are completely different diseases with different modes of transmission and disease course. First, HIV spreads through body fluids, while COVID-19 is currently considered an acute airborne infection [248]. People with chronic illnesses are at risk of relatively more severe COVID-19 symptoms, including those with weakened immune systems [249,250,251]. Therefore, HIV-1 infected patients may experience heightened feelings of anxiety about being infected with COVID-19 [248,252,253]. The US Centers for Disease Control and Prevention classifies immunocompromised people as high-risk, focusing on people living with uncontrolled HIV-1 or AIDS.

In addition to the unprecedented disruption of life, the COVID-19 pandemic has seriously hampered global HIV-1 care since the attention, resources, and personnel have been diverted to combat COVID-19 [254,255,256]. It is estimated that about 19% of HIV-infected patients could not receive antiretroviral drugs due to the pandemic. In addition, there have been reports that several HIV-1/AIDS prevention and control centers around the world have been converted to COVID-19 treatment centers, making it impossible for HIV-1 patients to receive therapy [257].

Surprisingly, some studies have shown that COVID-19 pathology is not very different in HIV-infected individuals compared to the general population [258,259,260]. HIV-positive patients do not develop the intense immunological response that often complicates the clinical course of COVID-19 [261]. Coinfection with HIV-1 and SARS-CoV-2 does not appear to cause a difference in clinical presentation. Currently, the COVID-19 guidelines for well-controlled HIV-1 infection indicate that it is unlikely that people living with HIV-1 are at greater risk of contracting COVID-19 or more severe illness than the general population [262].

Thus, the COVID pandemic has highlighted the need to develop new HIV-1 prevention and treatment approaches. Still, the Omicron variant, originally found in Botswana and South Africa, has made an even greater contribution. It was reported to the World Health Organization on 24 November 2021 and was identified as an option of concern on 26 November 2021. This outbreak of the Omicron variant in Europe and North America has shown that efficient vaccination may be powerless against new variants of the virus evolving in immunocompromised organisms. Undoubtedly, the Omicron variant is not the last example of how HIV-infected people can become reservoirs for new forms of diseases that might be dangerous for humanity.

Most people can effectively combat SARS-CoV-2 [263,264]. However, several reports suggest that long-term coronavirus infection can persist for many months in HIV-positive patients not receiving antiretroviral therapy [265,266]. It has been shown that the SARS-CoV-2, evolving in the patient for a long time, replicates and undergoes mutations, especially in the S-protein. It has been hypothesized that evolution within the host may be one of the mechanisms for the emergence of SARS-CoV-2 variants [267,268]. Thus, the emergence of new variants of SARS-CoV-2 in some cases may be associated with mutations occurring within HIV-1 infected patients with immunosuppression, with an advanced stage of HIV-1, which cannot get rid of SARS-CoV-2 [269].

Two cases of particular interest were identified in hospitals in South Africa [269]. One issue is devoted to the HIV-infected woman (with an advanced stage of HIV-1 and failure of antiretroviral treatment) at about 30 years with persistent infection with SARS-CoV-2. Despite a short clinical illness of moderate severity, a positive PCR result for SARS-CoV-2 lasted up to 216 days. Significant shifts in the viral population have been demonstrated during this time, including multiple mutations in key nAb epitopes in the RBD and N-terminal domain of spike protein [269]. In another documented case of long-term infection in an HIV-1 infected with profound immunodeficiency, genome sequencing showed only one mutation arising in the spike (T719I) at 53 days [270]. Most other cases of chronic COVID-19 infection were described in people with hematologic malignancies or people receiving immunosuppressive therapies for solid organ transplants or other chronic diseases [270,271]. Genomic and clinical data suggest that the evolution of the virus may have been triggered by selective pressure due to an impaired response of nAbs.

Two studies from South Africa demonstrated that HIV-1 is associated with suboptimal CD4^+^ T cells and humoral immune responses to SARS-CoV-2, especially in the absence of suppressive antiretroviral therapy [272,273]; and one US study showed that HIV-1 is associated with lower nAb titers after natural infection [274]. Immunosuppression due to the ineffectiveness of antiretroviral treatment and resistance to HIV-1 drugs leads to a violation of both cellular and adaptive humoral immunity, which prevents the elimination of SARS-CoV-2 [275].

South Africa has the world’s most extensive HIV-1 treatment program, with about 5.2 million people in therapy. Despite this, there remains a significant number of people with progressive HIV-1 infection [276,277]. Preliminary results in patients with controlled HIV-1 indicate that the immune response to COVID-19 vaccines is equivalent to that of HIV-negative people [278]. Still, more research is needed to understand the immunogenicity and effectiveness of vaccination and develop optimal dosing strategies, especially for patients with advanced HIV-1 infection. If the persistent infection does occur more frequently in the context of HIV-1, this could warrant a preference for people living with HIV-1 to be vaccinated against COVID-19.

We believe that concerns about the potential link between the emergence of new variants of infectious diseases in people living with HIV-1 will spur more active global action to develop HIV-1 vaccines, as HIV-1 prevention in many regions is not given adequate attention and funding due to the COVID-19 pandemic.

### 7.1. Immunological Features of COVID-19 in HIV-1 Infected Patients

If untreated, HIV-1 infection leads to a decrease in the CD4 T cell count, leading to AIDS. AIDS is defined as a CD4 cell count <200 cells/μL [279]. If HIV-1 infection is controlled, the risk of serious complications from COVID-19, and therefore poor outcomes, is likely to be low. However, the same cannot be said for poorly controlled HIV-1 infection or AIDS [280].

Some immunological features are inherent in both viral infections. Changes in lymphocyte subpopulations are characteristic of patients with COVID-19: lymphocyte depletion is a sign of severe COVID-19 [281,282,283]. An overall decrease in lymphocyte counts, including CD4^+^ T cells, CD8^+^ T cells, B cells, and NK cells, has been observed in severe and deceased patients with COVID-19 [284,285]. The acute phase of HIV-1 infection is characterized by a significant decrease in CD4^+^ T lymphocyte counts that persisted throughout the chronic phase, resulting in the lymphopenia seen in untreated AIDS patients [286,287,288].

Cytokine storm in patients with COVID-19 is associated with the severity of the outcome [289,290,291], HIV-1 infection is associated with persistent immune system impairment, even during effective antiretroviral therapy. This dysregulation may paradoxically prevent cytokine release in severe and critical COVID-19 [292,293]. This may explain the milder symptoms, lower morbidity, and lower mortality among HIV-infected patients infected with COVID-19 since the main fatal condition in COVID patients is caused by a cytokine storm, which subsequently leads to multiple organ dysfunction and death [294]. The lack of T cell activation is thought to mitigate the severe immunopathological events seen in COVID-19 [295]; the effects of the antiretroviral therapy were controversial. Antiretroviral therapy was proposed to protect against SARS-CoV-1 in 2003 [296]. Several antiretrovirals, including tenofovir and lopinavir, have shown antiviral activity against SARS-CoV-2 in vitro [297,298]. However, a randomized clinical trial of lopinavir/ritonavir showed no reduction in mortality in severe COVID-19 [299].

### 7.2. SARS-CoV-2 and HIV-1 Vaccines

Intensive research of HIV-1 and attempts to develop an HIV-1-vaccine at the end of the 20th century and of the early 21st century led to tremendous technical and scientific advances in virology, molecular immunology, and particularly vaccinology. These experimental results and clinical trials made it possible to develop effective vaccines against SARS-CoV-2 in the shortest time. Why were the SARS-CoV-2 vaccines produced so quickly, and the HIV-1 vaccine still is not yet developed? We suggest that the main difference between SARS-CoV-2 and HIV-1 is that the patients infected with SARS-CoV-2 can get rid of the virus. A double dose of vaccine stimulates a secondary immune response, which leads to the destruction of infected cells [264]. In the case of HIV-1, the virus integrates into the genome, forms a CD4^+^ T cell reservoir, leading to chronic infection [300]. The target of nAbs in the case of SARS-CoV-2 is the receptor-binding domain of the S-glycoprotein. Neutralizing Abs block the binding of SARS-CoV-2 virion to the Ace2-receptor [301,302,303]. Abs neutralizing SARS-CoV-2 are easily formed in the body after COVID-19 infection or after vaccination [304,305,306]; on the opposite, HIV-1 can evade immunity and possess significant genetic variation among strains. This poses serious obstacles to vaccine development; the vaccine must produce Abs that can adapt to the Env glycan screen and bind to various viral strains to provide protection [158,159].

## 8. Conclusions

The highest priority in HIV-1 research remains the development of a preventive vaccine. Despite many years of attempts to develop an HIV-1 vaccine based on classical strategies, it has not been possible so far. The search for a vaccine is still ongoing. One of the main focuses of a preventive vaccine is the induction of protective immune responses in the early stages of HIV-1 infection. Among the main obstacles encountered in developing an effective vaccine are mutational variability and global viral diversity, making it easy to avoid cellular and humoral host responses. One of the goals of studying Abs in HIV-1 infection is to determine how to safely induce broadly reactive protective Abs against HIV-1. At the same time, such Abs should trigger a cascade of autoimmune reactions. Since HIV-1 is transmitted primarily through mucous membranes, understanding antiviral immunity in mucosal sites is of great importance.

The first step in developing effective vaccines is understanding HIV-1 evolutionary strategies. One of these strategies is the ability of HIV-1 to suppress the adaptive immune response to vulnerable envelope epitopes, which must be maintained in the most conservative form since they are necessary to maintain the infectivity of the virus. Because of HIV-1’s ability to elude Ab responses, the vaccine must induce the production of multiple bNAbs that will target various conserved sites on the Env glycoprotein. Thus, several batches of immunogens may be required.

Various Abs against antigens were described in HIV-1, including autoAbs with catalytic activities. Biological functions of natural Abs in HIV-1 infection, and in the first place, polyreactive and autoAbs, have not been sufficiently studied. Thus, the task of analyzing natural and artificial Abs against HIV-1 in the context of developing an HIV-1 vaccine is unlikely to be completed soon. When creating a vaccine against HIV-1, it is necessary to consider the design of the immunogen and the method of its delivery, and the production of nAbs and avoid the production of harmful autoAbs. Among the priorities is the solution of the following problems: generation of bNAbs after vaccination and production of monoclonal bNAbs with broad prospects for therapeutic use.

## Figures and Tables

**Table 1:** The catalytic activity of natural Abs isolated from the blood serum of HIV-1-infected patients, their substrates, and individual epitopes.

Specific Substrate	Type of Abs (Ab Fragment)	Ref
gp120	L-chain, IgA	[136,137,138]
epitope 421–433 of gp120	IgM, IgA, L-chain	[136,137,141]
HIV-1 integrase	IgG, IgM	[42,43]
HIV-1 integrase oligopeptides	IgG, IgM	[133,143]
HIV-1 reverse transcriptase	IgG, IgM	[39]
HIV-1 reverse transcriptase oligopeptides	IgG, IgM	[134]
β-casein	IgG	[39]
DNA	IgG, L-chain	[39,152]
histones	IgG	[40]
MBP	IgG	[41]
MBP oligopeptides	IgG	[144]
short non-specific oligopeptides	IgG	[133]

**Table 2:** Completed HIV-1 vaccine studies according to the clinicaltrials.gov website.

Title	Conditions	ID
HIV Testing & Womens Attitudes on HIV Vaccine Trials	HIV-1	NCT00771537
Efficacy and Safety of GSK Biologicals HIV Vaccine in Antiretroviral Therapy (ART)-naïve HIV Infected Persons	HIV-1	NCT01218113
Follow up of Thai Adult Volunteers With Breakthrough HIV Infection After Participation in a Preventive HIV Vaccine Trial	HIV-1	NCT00337181
Study to Optimize the Quality of Samples for Cell-mediated Immunity (CMI) in ART-naïve HIV-infected Subjects	HIV-1	NCT01610427
HIV Vaccine Trial in Thai Adults	HIV-1	NCT00223080
A Safety and Immune Response Study of 2 Experimental HIV Vaccines	HIV-1	NCT02404311
Safety and Immunogenicity of Clade C ALVAC and gp120 HIV Vaccine	HIV-1	NCT03284710
The Safety and Immunogenicity of a Potential HIV Vaccine	HIV-1	NCT01966900
Investigation of V520 in a HIV Vaccine Dose Refinement Study (V520-027)	HIV-1	NCT00350623
Investigation of V520 in an HIV Vaccine Proof-of-Concept Study (V520-023)	HIV-1	NCT00095576
Dose-ranging Study to Evaluate the Safety & Immunogenicity of a HIV Vaccine 732,461 in Healthy HIV Seronegative Volunteers	HIV-1	NCT00434512
Effectiveness of Two Hepatitis B Vaccines in HIV-negative Youths	Hepatitis B	NCT00107042
A Pilot Study of a Dendritic Cell Vaccine in HIV-1 Infected Subjects	HIV-1	NCT00833781
HIV-1 Peptide Immunization of Individuals in West Africa to Prevent Disease	HIV-1	NCT01141205
Therapeutic Vaccine for HIV	HIV-1	NCT01859325
Safety and Immunogenicity of Anti-Pneumococcal Vaccines in HIV-Infected Pregnant Women	Pneumococcal	NCT02717494
H1N1 Influenza Vaccine Immunogenicity in HIV-1 Infected Patients	HIV-1	NCT01111162
Impact of a Human Papilloma Virus (HPV) Vaccine in HIV-Infected Young Women	HIV-1	NCT00710593
Safety and Effectiveness of HIV-1 DNA Plasmid Vaccine and HIV-1 Recombinant Adenoviral Vector Vaccine in HIV-Uninfected, Circumcised Men and Male-to-Female (MTF) Transgender Persons Who Have Sex With Men	HIV-1	NCT00865566
A Study of Safety, Tolerability, and Immunogenicity of the MRKAd5 Gag/Pol/Nef Vaccine in Healthy Adults (V520-016)	HIV-1	NCT00849680
Improving Immunogenicity of Influenza Vaccine in HIV Infected Individuals	HIV-1	NCT01262846
Immunogenicity of Hepatitis B Vaccination in HIV-infected Adults	Hepatitis B	NCT03316807
Immune Responses to Pneumococcal Vaccination Among HIV-infected Subjects	HIV-1	NCT00706550
Long-term Immunogenicity of the HIV gp120-NefTat/AS01B Vaccine (GSK SB732461)	HIV-1	NCT03368053
Early Versus Delayed BCG Vaccination of HIV-exposed Infants	HIV-1	NCT00712530
Combination Vaccination Before HIV Treatment Interruption	HIV-1	NCT02062580
Evaluating the Immunogenicity of the AIDSVAX B/E Vaccine and the MVA/HIV62B Vaccine in Healthy, HIV-1-Uninfected Adults Who Previously Received MVA/HIV62B in DNA/MVA or MVA/MVA Regimens in HVTN 205	HIV-1	NCT02852005
Evaluation of Safety and Immunogenicity of a Human Papillomavirus (HPV) Vaccine in Human Immunodeficiency Virus (HIV) Infected Females	HPV-1	NCT01031069
Immunogenicity of Fluzone HD, A High Dose Influenza Vaccine, In Children With Cancer or HIV	HIV-1	NCT01205581
Shedding, Immunogenicity and Safety of Quadrivalent Live Intranasal Influenza Vaccine (QLAIV) in HIV-infected Children and Young Adults	HIV-1	NCT02474901
Live Zoster Vaccine in HIV-Infected Adults on Antiretroviral Therapy	Herpes Zoster	NCT00851786
Safety of and Immune Response to an H1N1 Influenza Virus Vaccine in HIV Infected Children and Youth	H1N1 Influenza	NCT00992836
Safety and Immune Response of a Rotavirus Vaccine in HIV-infected and Uninfected Children Born to HIV-infected Mothers	Rotavirus	NCT00880698
Safety and Immunogenicity of 13-Valent Pneumococcal Conjugate Vaccine (13vPnC) in HIV-Infected Subjects 6 Years of Age or Older Who Are € to Pneumococcal Vaccine	Pneumococcal	NCT00962780
Safety of and Immune Response to an H1N1 Influenza Vaccine in HIV Infected Pregnant Women	H1N1 Influenza	NCT00992017
Immunization With HIV-1 Peptides in Adjuvant for Treatment of Patients With Chronic HIV-infection	HIV-1	NCT01009762
Safety and Immunogenicity of IMVAMUNE (MVA-BN) Smallpox Vaccine in HIV Infected Patients	HIV-1	NCT00316589
Autologous Dendritic Cell Vaccine in HIV1 Infection	HIV-1	NCT00510497
Quadrivalent HPV Vaccine to Prevent Anal HPV in HIV-infected Men and Women	HIV-1	NCT01461096
Safety of and Immune Response to the Human Papillomavirus (HPV) Vaccine in HIV-Infected Women	HPV-1	NCT00604175
Comparison of Three Hepatitis B Vaccination Regimens in HIV-Positive Youth	Hepatitis B	NCT00106964
Safety of and Immune Response to a Novel Human Papillomavirus Vaccine in HIV Infected Children	HPV	NCT00339040
Safety of and Immune Response to a Meningitis Vaccine in HIV Infected Children and Youth	Meningitis	NCT00459316
Safety and Efficacy of Romidepsin and the Therapeutic Vaccine Vacc-4x for Reduction of the Latent HIV-1 Reservoir	HIV-I	NCT02092116
Safety and Immunogenicity of GlaxoSmithKline Biologicals’ HPV Vaccine 580,299 (Cervarix) in HIV Infected Females	HPV	NCT00586339
The Effect of HPV Vaccination on Recurrence Rates in HIV Patients With Condylomata	HPV	NCT00941889
Immune Response After Booster Vaccination in HIV-Infected Patients Who Received Rabies Primary Vaccination	Rabies	NCT01286493
Safety and Immunogenicity of a Candidate Tuberculosis (TB) Vaccine in HIV-positive Adults.	Tuberculosis	NCT00707967
Safety & Immunogenicity of 13vPnC in HIV-Infected Subjects Aged 18 or Older Who Were Previously Immunized With 23vPS	Pneumococcal	NCT00963235
Study to Evaluate GSK Biologicals’ Herpes Zoster Vaccine GSK1437173A in Human Immunodeficiency Virus HIV Infected Subjects	Herpes Zoster	NCT01165203
Vaccine Therapy in Preventing HPV in HIV-Positive Women in India	HPV	NCT00667563
Evaluation of the Immune Response of a HIV Candidate Vaccine After Administration of One Chloroquine Dose	HIV-1	NCT00972725
Evaluating the Safety and Immunogenicity of HIV Clade C DNA Vaccine and MF59- or AS01B-Adjuvanted Clade C Env Protein Vaccines in Various Combinations in Healthy, HIV-Uninfected Adults	HIV-1	NCT02915016
Safety and Immunogenicity of GSK Biologicals’ Investigational Malaria Vaccine in HIV Infected Infants and Children	Malaria	NCT01148459
Magnitude of the Antibody Response to and Safety of a GBS Trivalent Vaccine in HIV Positive and HIV Negative Pregnant Women and Their Offsprings	Streptococcal	NCT01412801
Vaccine Therapy in Preventing Human Papillomavirus Infection in Young HIV-Positive Male Patients Who Have Sex With Males	HPV	NCT01209325
Primary and Booster Vaccination Study With a Pneumococcal Vaccine in HIV Infected, HIV Exposed Uninfected and HIV Uninfected Children 6 to 10 Weeks of Age.	Pneumococcal	NCT00829010
Intradermal Versus Intramuscular Polio Vaccine Booster in HIV Infected Subjects	Polio	NCT01686503
Human Papillomavirus Vaccine Therapy in Treating Men With HIV-1 Infection	HPV	NCT00513526
MRKAd5 HIV-1 Gag Vaccine (V520) in Subjects With Chronic Hepatitis C (V520-022)	Hepatitis C	NCT00857311
